# *LifeSnaps*, a 4-month multi-modal dataset capturing unobtrusive snapshots of our lives in the wild

**DOI:** 10.1038/s41597-022-01764-x

**Published:** 2022-10-31

**Authors:** Sofia Yfantidou, Christina Karagianni, Stefanos Efstathiou, Athena Vakali, Joao Palotti, Dimitrios Panteleimon Giakatos, Thomas Marchioro, Andrei Kazlouski, Elena Ferrari, Šarūnas Girdzijauskas

**Affiliations:** 1 Aristotle University of Thessaloniki, School of Informatics, Thessaloniki, 54124 Greece; 2 Earkick, Zurich, 8008 Switzerland; 3 Foundation for Research and Technology Hellas, Heraklion, 70013 Greece; 4 University of Insubria, Varese, 21100 Italy; 5 KTH Royal Institute of Technology, Stockholm, 11428 Sweden

**Keywords:** Genetic databases, Scientific data, Computer science, Information technology

## Abstract

Ubiquitous self-tracking technologies have penetrated various aspects of our lives, from physical and mental health monitoring to fitness and entertainment. Yet, limited data exist on the association between in the wild large-scale physical activity patterns, sleep, stress, and overall health, and behavioral and psychological patterns due to challenges in collecting and releasing such datasets, including waning user engagement or privacy considerations. In this paper, we present the **LifeSnaps dataset**, a multi-modal, longitudinal, and geographically-distributed dataset containing a plethora of anthropological data, collected unobtrusively for the total course of more than 4 months by *n* = 71 participants. LifeSnaps contains more than 35 different data types from second to daily granularity, totaling more than 71 M rows of data. The participants contributed their data through validated surveys, ecological momentary assessments, and a Fitbit Sense smartwatch and consented to make these data available to empower future research. We envision that releasing this large-scale dataset of multi-modal real-world data will open novel research opportunities and potential applications in multiple disciplines.

## Abstract (summary)

**Table:** 

Measurement(s)	Step Unit of Distance • Nutrition, Calories • Physical Activity Measurement • Oxygen Saturation Measurement • maximal oxygen uptake measurement • Electrocardiography • Respiratory Rate • skin temperature sensor • Unit of Length • Very Light Exercise • heart rate variability measurement • resting heart rate • electrodermal activity measurement • State-Trait Anxiety Inventory • Positive and Negative Affect Schedule • Stages and Processes of Change • Behavioural Regulations in Exercise • 50-item International Personality Item Pool version of the Big Five Markers
Technology Type(s)	FitBit • Survey • Survey
Sample Characteristic - Organism	Homo sapiens
Sample Characteristic - Environment	anthropogenic habitat
Sample Characteristic - Location	Greece • Italy • Sweden • Cyprus

## Background & Summary

The past decade has witnessed the arrival of ubiquitous wearable technologies with the potential to shed light on many aspects of human life. Studies with toddlers^1^, older adults^2^, students^3^, athletes^4^, or even office workers^5^ can be enriched with wearable devices and their increasing number of measured parameters. While the use of wearable technologies fosters research in various areas, such as privacy and security^6^, activity recognition^7^, human-computer interaction^8^, device miniaturization^9^ and energy consumption^10^, one of the most significant prospects is to transform healthcare by providing objective measurements of physical behaviors at a low-cost and unprecedented scale^11^. Many branches of medicine, such as sports and physical activity^12^, sleep^13^ and mental health^14^, to name a few, are turning to wearables for scientific evidence. However, the laborious work of clinically validating the biometric data generated by these new devices is of critical importance and often disregarded^15^.

In recent years, with the intent to further clinically validate the data generated by smart and wearable devices, considerable effort has been directed toward building several datasets covering a variety of health problems and sociological issues^3,16–21^. For instance, the largest cohort ever studied comes from Althoff *et al*.^16^ in 2017. They collected 68 M days of physical activity data from 717k mobile phone users distributed across 111 countries. The study aimed to correlate inactivity and obesity within and across countries. Unfortunately, the data from this study is somewhat limited to any other research question, as only the aggregated numbers were made public. Likewise, exclusively focusing on asthma, Chan *et al*.^17^ also developed a mobile application to collect data from 6k users with daily questionnaires on the participants’ asthma symptoms. With a focus on cardiovascular health, the MyHeartCounts Cardiovascular Health Study^18^ is another extensive study containing 50k participants recruited in the US within six months. Although many participants enrolled in the study, the mean engagement time with the app per user was only 4.1 days. As a result of selecting only one health problem and collecting data exclusively for that specific condition, the collected data are often of limited use to other researchers. Closer to our scale and philosophy, Philip Schmidt *et al*.^21^ published the WESAD dataset, studying stress among 15 people, recent graduates, in the lab during a 2-hour experiment. Thus, WESAD is inherently different as it does not capture a realistic picture of our lives in the wild, given that the recruiting is restricted, and the emotional conditions are provoked in lab conditions. Similar to our study, Vaizman *et al*.^20^ collected data from 60 users, exploiting different types of everyday devices like smartphones and smartwatches. Nevertheless, the mean participation time was only 7.6 days. In the same line, the StudentLife^3^ app collected daily data from the devices of 48 students throughout the 10-week term at Dartmouth College, seeking the effect of workload on stress levels and general students’ well-being and mental health. Table 1 facilitates the comparison between the contributions and limitations of the above-mentioned previous works. Our study possesses complementary contributions, incorporating new sensors and surveys, and subsequently adding new data types overtaking the above-mentioned works.

**Table 1:** Comparison between related datasets and the LifeSnaps data.

Datasets	Contributions						
	Open to use data	Multimodal data	Novel datatypes	Rich granularity	In-the-wild data	Interdisciplinarity	Reproducibility
Activity inequality^16^				✓	✓	✓	
The asthma mobile health study^17^	✓	✓			✓		
MyHeart Counts Study^18^	✓	✓					
WESAD^21^	✓	✓					✓
Recognizing Detailed Human Context In-The-Wild^20^	✓	✓	✓		✓	✓	
StudentLife^3^	✓	✓			✓		
**LifeSnaps**	✓	✓	✓	✓	✓	✓	✓

**Fig. 1:**
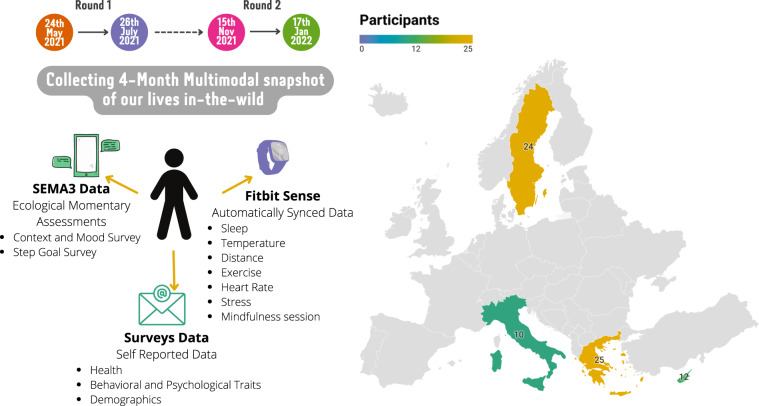
The study timeline, the data collected, and the country of residency of our participants in Europe.

Our goal is to provide researchers and practitioners with a rich, open-to-use dataset that has been thoroughly anonymized and ubiquitously captures human behavior in the wild, namely humans’ authentic behavior in their regular environments. Designed to be of general utility, this paper introduces the **LifeSnaps dataset**, a multi-modal, longitudinal, space and time distributed dataset containing a plethora of anthropological data such as physical activity, sleep, temperature, heart rate, blood oxygenation, stress, and mood of 71 participants collected from mid-2021 to early-2022. Figure 1 summarizes the study in terms of modalities, locations, and timelines.

In synthesis, the LifeSnaps dataset’s main contributions are:

- **Privacy and anonymity:** we follow the state-of-the-art anonymization guidelines^22^ and EU’s General Data Protection Regulation (GDPR)^23^ to collect, process, and distribute participants’ data, and ensure privacy and anonymity for those involved in the study. Specifically, the LifeSnaps study abides by GDPR’s core principles, namely lawfulness, fairness and transparency, purpose limitation, data minimization, accuracy, storage limitation, and integrity and confidentiality^23^.
- **Data modalities:** we use three complementary, heterogeneous data modalities: (1) survey data, containing evaluations such as personality and anxiety scores; (2) ecological momentary assessments to extract participants’ daily goals, mood, and context; and (3) sensing data from a flagship smartwatch;
- **Emerging datatypes:** due to the use of a recently released device (FitBit Sense was released at the end of September 2020), various new data types that are rarely studied, such as temperature, oxygen saturation, heart rate variability, automatically assessed stress and sleep phases are now open and available, empowering the next wave of sensing research;
- **Rich data granularity:** we aim to provide future researchers with the best data granularity possible, for that, we make available data as raw as technologically possible. Different data types exhibit different levels of detail, from second to daily granularity, always with respect to privacy constraints;
- **Community code sharing and reproducibility**: apart from the raw data format, we also provide a set of preprocessing scripts (https://github.com/Datalab-AUTH/LifeSnaps-EDA), aiming to facilitate future research and familiarize researchers and practitioners who can largely exploit and use LifeSnaps data;
- **In the wild data**: participants were explicitly told to move on with their lives, as usual, engaging in their natural and unscripted behavior, while no lab conditions or restrictions were imposed on them. This ensures the in the wild nature of the dataset and subsequently its ecological validity;
- **Trade-offs scale, duration and participation:** we designed the study to have a fair balance between the study length (8 weeks) and the number of participants (71), setting and conducting weekly reminders and follow-ups to maintain engagement. For this reason, we envision the LifeSnaps dataset can facilitate research in various scientific domains.

Our goal in sharing LifeSnaps is to empower future research in different disciplines from diverse perspectives. We believe that LifeSnaps, as a multi-modal, privacy-preserving, distributed in time and space, easily reproducible, and rich granularity time-series dataset will help the scientific community to answer questions related to human life, physical and mental health, and overall support scientific curiosity concerning sensing technologies.

## Methods

This section discusses participants’ recruitment and demographics and moves on to describe the study’s timeline, data collection process, and data availability. Finally, it sheds light on user engagement, data quality, and completeness issues.

### Participants

Participants’ recruitment was geographically distributed in four countries, namely Greece, Cyprus, Italy, and Sweden, while the study was approved by the Institutional Review Board (IRB) of the Aristotle University of Thessaloniki (Protocol Number 43661/2021). All study participants were recruited through convenience sampling or volunteering calls to university mailing lists. In the end, the locations of the participants recruited coincide with the locations of the partner universities of the RAIS consortium, which organized the LifeSnaps study. Namely, there were 24 participants in Sweden, 10 in Italy, 25 in Greece, and 12 in Cyprus, as depicted in Fig. 1. Participants were not awarded any monetary or other incentives for their participation. As mentioned earlier, all participants provided written informed consent to data processing and to de-identified data sharing. The content of the informed consent is also delineated on the study’s web page (https://rais-experiment.csd.auth.gr/the-experiment/). Specifically, released data is de-identified and linked only by a subject identifier. We initially recruited 77 participants, of which 4 dropped out of the study, 1 faced technical difficulties during data export, and 1 withdrew consent, leading to a total number of 71 study participants. This translates to a 7% drop rate, lower than similar in the wild studies^3,18^. We further discuss user engagement with the study in a later section.

To participate, subjects were required to (a) have exclusive access to a Bluetooth-enabled mobile phone with internet access without any operating system restrictions for the full study duration, (b) have exclusive access to a personal e-mail for the full study duration, (c) be at least 18 years old at the time of recruitment, and (d) be willing to wear and sync a wearable sensor so that data can be collected and transmitted to the researchers.

**Fig. 2:**
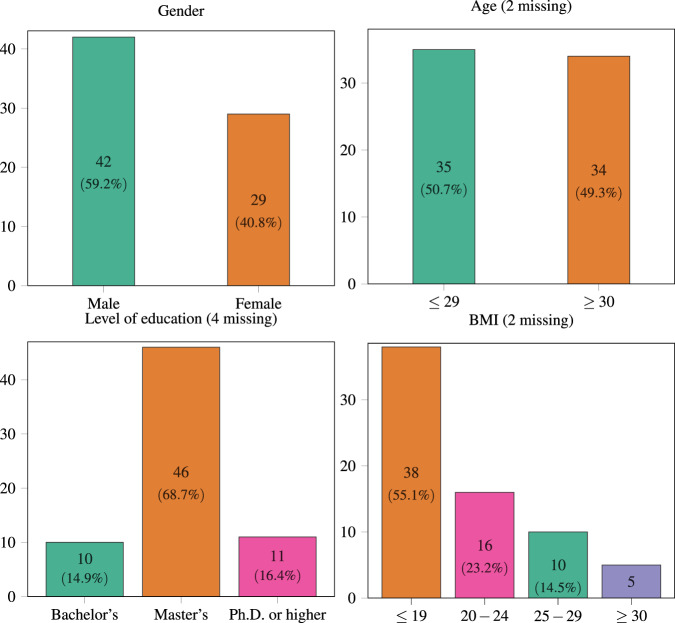
Histograms representing the distributions of demographic attributes among the 71 participants. Each column displays the number of occurrences for the corresponding category. Note that some participants did not reveal their age, BMI, or level of education.

Among the 71 participants who completed the study, 42 are male, and 29 are female, with an approximate 60/40 ratio. All but two provided their age, half under 30 and half over 30 (ranges are defined so that k-anonymity is guaranteed). Two participants reported incorrect weight values, making it impossible to compute their BMI. Among the rest, 55.1% had a BMI of 19 or lower, 23.2% were in the range 20–24, 14.5% were in the range 25–29, and the remaining 7.2% had 30 or above. The highest level of education for most participants (68%) is the Master’s degree, while 14.9% had completed Bachelor’s and the other 16.4% had a Ph.D. or a higher degree. Histograms summarizing these distributions are shown in Fig. 2.

With regard to the participants’ usage of wearable devices, out of 66 responses, 51% stated that they currently own or have owned a wearable device in the past. Out of those who have not used wearables, 40% identified high cost as a preventive factor, while only 3% mentioned technical difficulties or trust in their own body, and none reported general mistrust towards wearables (multiple choice possible). Out of those who do use wearables, 44% purchased them to gain increased control over their physical activity, 44% due to general interest in technology, 38% for encouragement reasons, 35% for health monitoring, 18% received them as a gift, 15% after a doctor’s recommendation, and 9% through an insurance rewards’ program (multiple choice possible). The most common indicators tracked include heart rate (91%), step count (88%), distance (73%), calories (62%), and exercise duration (55%). Finally, concerning sharing of sensing data, 32% of participants have shared data with researchers or friends, or family, while a smaller percentage has shared data with exercise platforms (15%), such as Strava, or their doctor (6%).

All study participants wore a Fitbit Sense smartwatch (https://www.fitbit.com/global/us/products/smartwatches/sense) for the duration of the study, while no other incentives were given to the participants. The watch was returned at the end of the study period.

### Study procedure

**Fig. 3:**
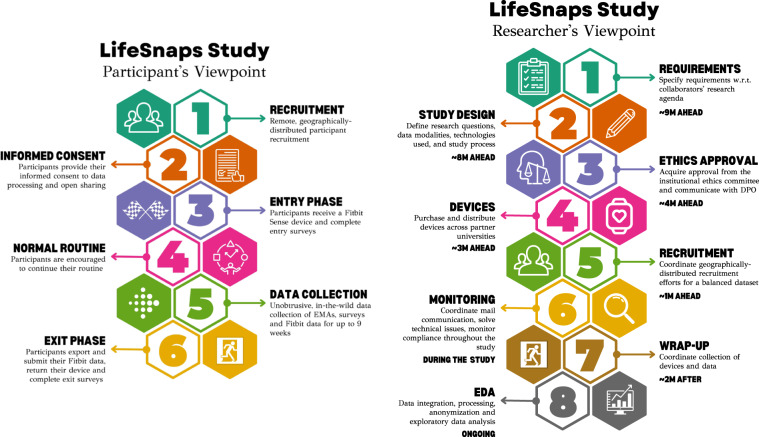
The study procedure from both the participants (left) and researchers’ (right) viewpoint.

The LifeSnaps Study is a two-round study; the first round ran from May 24th, 2021 to July 26th, 2021 (*n* = 38), while the second round ran from November 15th, 2021 to January 17th, 2022 (*n* = 34), totaling more than four months and 71 M rows of user data. One participant joined both study rounds. In this section, we discuss the entry and exit procedure, the data collection and monitoring, and related privacy considerations (see Fig. 3).

#### Participant entry and exit

Upon recruitment, each participant received a starter pack, including information about the study (e.g., study purpose, types of data collected, data management), as well as a step-by-step instructions manual (https://rais-experiment.csd.auth.gr/). Note that we conducted the study amidst the Covid-19 pandemic in compliance with the national and regional restrictions, minimizing in-person contact and encouraging remote recruitment. Initially, each participant provided written informed consent to data processing and sharing of the de-identified data. Specifically, they were informed that after the end of the study, the data would be anonymized so that their identity could no longer be inferred and shared with the scientific community. Then, they were instructed to set up their Fitbit devices, authorize our study compliance app to access their Fitbit data, and install the SEMA3 EMA mobile application^24^. Participants were encouraged to carry on with their normal routine and wear their Fitbit as much as possible. At the end of the study, participants were instructed to export and share their Fitbit data with the researchers and reset and return their Fitbit Sense device.

**Fig. 4:**
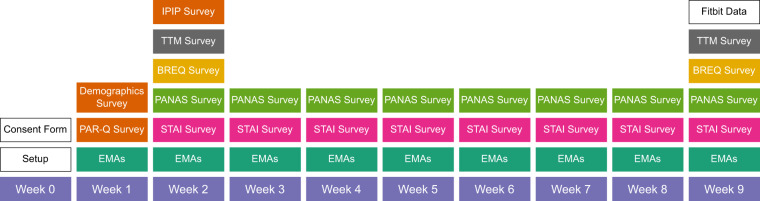
Time flow of the 9-week study.

During the entry stage, a series of health, psychological, and demographic baseline surveys were administered using a university-hosted instance of LimeSurvey^25^. During the exit stage, we re-administered a subset of the entry surveys as post-measures. Specifically, we distributed a demographics survey, the Physical Activity Readiness Questionnaire (PAR-Q)^26^, and the 50-item International Personality Item Pool version of the Big Five Markers (IPIP)^27^ at entry, and the Stages and Processes of Change Questionnaire^28,29^, a subset of the transtheoretical model (TTM) constructs^30,31^, and the Behavioural Regulations in Exercise Questionnaire (BREQ-2)^32,33^ at entry and exit, as shown in Table 2. Note that the demographics and PAR-Q surveys are excluded from the published dataset for anonymity purposes. Figure 4 delineates the entry and exit stages’ flow.

**Table 2:** The three data modalities in the LifeSnaps Dataset: Surveys, SEMA3, and Fitbit data, accompanied by data types, a short description, and related statistics.

Modality	Data Type	Description	First Appears	Recurring	#unique users	#responses
Surveys	Demographics Questionnaire	Survey w.r.t. gender, age, ethnicity, education, and usage of wearable devices (partly excluded for anonymization purposes)	Week 1	Entry	66	66
	Physical Activity Readiness Questionnaire (PAR-Q)^26^	Survey to determine the safety or possible risks of exercising based on health history, current symptoms, and risk factors (excluded for anonymization purposes)	Week 1	Entry	57	N/A
	Stages and Processes of Change Questionnaire^28,29^	Survey to determine dimensions of the Transtheoretical model^31^ that enable us to understand how shifts in exercise behavior occur	Week 2	Entry-Exit	55	102
	Behavioural Regulations in Exercise Questionnaire (BREQ-2)^32^	Survey to measure the stages of the self-determination continuum^33^ with respect to motivation to exercise	Week 2	Entry-Exit	54	99
	50-item International Personality Item Pool version of the Big Five Markers (IPIP)^27^	Survey to assess users on the Big Five model of Openness, Conscientiousness, Extraversion, Agreeableness & Neuroticism^55^	Week 2	Entry	52	52
	Positive and Negative Affect Schedule Questionnaire (PANAS)^44^	Survey to measure an individual’s positive and negative affect	Week 3	Weekly	53	302
	State-Trait Anxiety Inventory (S-STAI)^45^	Survey to assess trait anxiety in individuals	Week 3	Weekly	55	314
SEMA3	Context and Mood Survey	EMA survey to collect data on an individual’s context (location) and mood	Week 1	Thrice Daily	63	11526
	Step Goal Survey	EMA survey to determine an individual’s daily step goal	Week 1	Daily	61	3852
Fitbit	ECG	An electrocardiogram (ECG) is a test that measures the electrical activity of the heartbeat	Week 1	Arbitrary	15	73
	SpO2	Blood oxygen saturation (SpO2) is the percentage of your blood that’s saturated with or contains oxygen	Week 1	Daily	27	1274
	Respiratory Rate Summary	Breathing rate, also known as respiratory rate, is the number of breaths you take per minute.	Week 1	Daily	44	3000
	Estimated Oxygen Variation	Estimated oxygen variation (EOV) is an approximation of the changes in your blood oxygen saturation levels	Week 1	2-Minute	71	2009637
	VO2 max	VO2 max is a measure of the maximum amount of oxygen the body can utilize during exercise	Week 1	2-Day	71	4854
	Nightly (Computed) Temperature	Skin temperature variation from a 3-day baseline temperature, measured during nighttime	Week 1	Daily	67	3568
	Wrist Temperature	Skin temperature is the temperature on the skin’s surface, measured during nighttime at regular intervals	Week 1	3-Minute	61	4372238
	Stress Score	Stress score estimates how the body responds to stress based on heart rate, sleep, and activity level data	Week 1	Daily	36	1911
	Distance	Distance walked by an individual	Week 1	3-Minute	68	3010529
	Calories	Number of calories burned by an individual	Week 1	3-Minute	71	9675782
	Sleep	Sleep schedule, minutes asleep and awake, time spent in bed, time spent in each sleep stage	Week 1	Daily	66	4141
	Steps	Number of steps walked by an individual	Week 1	3-Minute	68	3010529
	Exercise	Types and duration of exercises performed by an individual	Week 1	Arbitrary	69	3275
	Active Minutes	Time spent in activity calculated through metabolic equivalents (METs)	Week 1	Daily	71	21609
	Sedentary Minutes	Time spent in sedentariness	Week 1	Daily	71	7203
	Time in Heart Rate Zones	Time spent in the fat burn, cardio, or peak heart-rate zones	Week 1	2-Day	71	4876
	Altitude	Number of floors climbed by the user	Week 1	Arbitrary	71	81022
	Heart Rate Variability	Heart rate variability (HRV) is the variation of time between each heartbeat	Week 1	5-Minute	44	225987
	Heart Rate	Heart rate is the speed of the heartbeat measured by the number of contractions of the heart per minute.	Week 1	< 1-minute	69	48720040
	Resting Heart Rate	Resting heart rate is the number of times the heart beats per minute when at rest	Week 1	4-Day	71	12362
	Profile	Demographic details about an individual	N/A	N/A	70	70
	Badge	Rewards earned for completing daily and lifetime milestones	Week 1	Arbitrary	71	902
	Journal Entries (Mood)	Manual mood entries after mindfulness sessions	Week 1	Arbitrary	46	118
	Mindfulness EDA	Electrodermal activity (EDA) responses are small changes in the sweat level of the skin	Week 1	<1-Minute	39	16070
	Mindfulness Goals	An individual’s goal number of mindfulness sessions to achieve	Week 2	Arbitrary	46	30
	Mindfulness Sessions	Mindfulness sessions can help the individual track and understand the effects of mindfulness practice	Week 1	Arbitrary	44	143

### Data acquisition

The data collection phase lasted for up to 9 weeks for each study round, incorporating three distinct data modalities, each with its own set of advantages as discussed below (see Fig. 4 for the time flow of the 9-week study):

#### Fitbit data

This modality includes 71 M rows of sensing data collected in the wild from the participants’ Fitbit Sense devices. The term “in the wild” was first coined in the field of anthropology^34–36^, referring to cognition in the wild. Nowadays, its definition is broader, referring to “research that seeks to understand new technology interventions in everyday living”^37^. Sensing data collected in the wild are likely to provide a more accurate and representative picture of an individual’s behavior than a snapshot of data collected in a lab^38^, thus facilitating the development of ecologically valid systems that work well in the real world^39^.

At the time of the study, Fitbit Sense is the flagship smartwatch of Fitbit embedded with a 3-axis accelerometer, gyroscope, altimeter, built-in GPS receiver, multi-path optical heart rate tracker, on-wrist skin temperature, ambient light, and multipurpose electrical sensors, as per the device manual and sensor specifications^40^. While the majority of offline raw sensor data (e.g., accelerometer or gyroscope measurements) are not available for research purposes, we have collected a great number of heterogeneous aggregated data of varying granularity, as shown in Table 2. Such data include, but are not limited to, steps, sleep, physical activity, and heart rate, but also data types from newly-integrated sensors, such as electrodermal activity (EDA) responses, electrocardiogram (ECG) readings, oxygen saturation (SpO2), VO2 max, nightly temperature, and stress indicators as shown in Table 2. All timestamp values follow the form of the Fitbit API as mentioned in the documentation (https://dev.fitbit.com/build/reference/device-api/accelerometer/), while all duration values in Fitbit data types, such as exercise, mindfulness sessions, and sleep types, are measured in milliseconds.

#### SEMA3 data

This modality includes more than 15 K participants’ Ecological Momentary Assessment (EMA) self-reports with regard to their step goals, mood, and location. EMA involves “repeated sampling of subjects’ current behaviors and experiences in real-time, in subjects’ natural environments”^41^. EMA benefits include more accurate data production than traditional surveys due to reduced recall bias, maximized ecological validity, and the potential for repeated assessment^41^. Additionally, complementary to sensing data that do not comprehensively evaluate an individual’s daily experiences, EMA surveys can capture more detailed time courses of a person’s subjective experiences and how they relate to objectively measured data^42^.

We distribute two distinct EMAs through the SEMA3 app: the step goal EMA (from now onward called Step Goal Survey) and the context and mood EMA (from now onward called Context and Mood Survey), as seen in Table 2. Once a day (morning), we schedule the Step Goal Survey; namely, we ask the participants how many steps they would like to achieve on that day with choices ranging from less than 2000 to more than 25000. Thrice a day (morning-afternoon-evening), we schedule the Context and Mood Survey, namely, we inquire the participants about their current location and feelings. The location choices include home, work/school, restaurant/bar/entertainment venues, transit, and others (free text field). An option “Home Office” is also added on the second round upon request. The mood choices include happy, rested/relaxed, tense/anxious, tired, alert, and sad, while an option neutral is added at the beginning of the first round upon request.

#### Surveys data

This modality includes approximately 1 K participants’ responses to various health, psychological and demographic surveys administered via Mailchimp, an e-mail marketing service (https://mailchimp.com/). Surveys have the benefit of accurately capturing complex constructs which would otherwise be unattainable, such as personality traits, through the usage of validated instruments. A validated instrument describes a survey that has been “tested for reliability, i.e., the ability of the instrument to produce consistent results and validity, i.e., the ability of the instrument to produce correct results. Validated instruments have been extensively tested in sample populations, are correctly calibrated to their target, and can therefore be assumed to be accurate^43^.

Apart from the entry and exit surveys discussed above, namely demographics, PAR-Q, IPIP, TTM, and BREQ-2, participants received weekly e-mails asking them to complete two surveys: the Positive and Negative Affect Schedule Questionnaire (PANAS)^44^, and the State-Trait Anxiety Inventory (S-STAI)^45^. For more details on the administered surveys, refer to Table 2.

### Data acquisition monitoring

During the study, we adopted various monitoring processes that automatically produce statistics on compliance. If a participant’s Fitbit did not sync for more than 48 h, we would send an e-mail to the participant with a reminder and a troubleshooting guide, while also informing the research team. Also, if a participant’s response rate in the SEMA3 app fell below 50%, the researchers would get notified to ping the participants to try and get them back on track. Finally, survey completion rates and e-mail opens and clicks were also monitored for the entry and exit surveys to ensure maximum compliance. Re-sent e-mails were sent every Tuesday to those participants who did not open their start-of-the-week e-mail on Monday to remind them to complete their weekly surveys.

### Data preprocessing

Certain preprocessing steps were required to ensure data coherence as indicated below:

#### Modality-agnostic user ID

For linking the three distinct data modalities, namely Fitbit, SEMA3, and surveys data, we assign a 2-byte random id to each participant, which is common across all modalities to replace modality-specific user identifiers. This modality-agnostic user identifier enables data joins between different modalities.

#### Time zone conversion

Given the geographically-distributed nature of our study, we also need to establish a common reference time zone to facilitate analysis and between-user comparisons. Due to privacy considerations, we cannot disclose the users’ time zones or their UTC offset. Hence, we convert all data of sub-daily granularity from UTC to local time. Specifically, concerning Fitbit data, only a subset of types are stored in UTC (see Table 3), as also verified by the Fitbit community (https://community.fitbit.com/t5/Fitbit-com-Dashboard/Working-with-JSON-data-export-files/td-p/3098623). These types are converted to local time based on the participant’s time zone, as declared in their Fitbit profile. Similarly, we convert SEMA3 and survey data modalities from the export timezone to local time using the same methodology. We also consider Daylight Saving Time (DST) during the conversion, since all recruitment countries follow this practice at the time of the study.

**Table 3:** The export time zone of different Fitbit data types: some types are exported in UTC, while others in local time. Time zone does not apply to daily granularity data types.

Type	Daily Granuarity	UTC	Local
ECG		+	
SpO2	+		
Respiratory Rate Summary	+		
Estimated Oxygen Variation			+
VO2 max	+		
Nightly (Computed) Temperature			+
Wrist Temperature			+
Stress Score	+		
Distance		+	
Calories			+
Sleep			+
Steps		+	
Exercise		+	
Active Minutes	+		
Sedentary Minutes	+		
Time in Heart Rate Zones	+		
Altitude			+
Heart Rate Variability			+
Heart Rate		+	
Resting Heart Rate	+		
Badge	+		
Journal Entries (Mood)	+		
Mindfulness EDA		+	
Mindfulness Goals	+		
Mindfulness Sessions			+

#### Translations

Due to the participants’ multilingualism, the exported Fitbit data, specifically exercise types, are exported in different languages. To this end, and with respect to the users’ privacy, we translate all exercise types to their English equivalent based on the unique exercise identifier provided.

#### Tabular data conversion

To facilitate the reusability of the LifeSnaps dataset, additionally to the raw data, we convert Fitbit and SEMA3 data to a tabular format to store in CSV files (see later for code availability). During the conversion process, we read each data type independently and perform a series of processing steps, including type conversion for numerical or timestamped data (data are stored as strings in MongoDB), duplicate elimination for records with the same user id and timestamp, and, optionally, aggregation (average, summation, or maximum) for data types of finer than the requested granularity.

#### Surveys scoring

Similarly, to facilitate the integration of the survey data, we provide, additionally to the raw responses, a scored version of each survey (scale) in a tabular format (see later for code availability). Each survey has by definition a different scoring function as described below:

##### IPIP

For the IPIP scale, we follow the official scoring instructions (https://ipip.ori.org/new_ipip-50-item-scale.htm). Each of the 50 items is assigned to a factor on which that item is scored (i.e., of the five factors: (1) Extraversion, (2) Agreeableness, (3) Conscientiousness, (4) Emotional Stability, or (5) Intellect/Imagination), and its direction of scoring (+ or −). Negatively signed items are inverted. Once scores are assigned to all of the items in the 50-item scale, we sum up the values to obtain a total scale score per factor. For between-user comparisons, we also add a categorical variable per factor that describes if the user scores below average, average, or above average with regard to this factor conditioned on the user’s gender.

##### TTM

For the TTM scale, each user is assigned a stage of change (i.e., of the five stages: (1) Maintenance, (2) Action, (3) Preparation, (4) Contemplation, or (5) Precontemplation) based on their response to the respective scale. Regarding the Processes of Change for Physical Activity, each item is assigned to a factor on which that item is scored (i.e., of the 10 factors: (1) Consciousness Raising, (2) Dramatic Relief, (3) Environmental Reevaluation, (4) Self Reevaluation, (5) Social Liberation, (6) Counterconditioning, (7) Helping Relationships, (8) Reinforcement Management, (9) Self Liberation, or (10) Stimulus Control). Once scores are assigned to all of the items, we calculate each user’s mean for every factor, according to the scoring instructions (https://hbcrworkgroup.weebly.com/transtheoretical-model-applied-to-physical-activity.html).

##### BREQ-2

For the BREQ-2 scale, each item is again assigned to a factor on which that item is scored (i.e., of the five factors: (1) Amotivation, (2) External regulation, (3) Introjected regulation, (4) Identified regulation, (5) Intrinsic regulation). Once scores are assigned to all of the items, we calculate each user’s mean for every factor, according to the scoring instructions (http://exercise-motivation.bangor.ac.uk/breq/brqscore.php). We also create a categorical variable describing the self-determination level for each user, namely the maximum scoring factor.

##### PANAS

For the PANAS scale, each item contributes to one of two affect scores (i.e. (1) Positive Affect Score, or (2) Negative Affect Score). Once items are assigned to a factor, we sum up the item scores per factor as per the scoring instructions (https://ogg.osu.edu/media/documents/MB%20Stream/PANAS.pdf). Scores can range from 10 to 50, with higher scores representing higher levels of positive or negative affect, respectively.

##### S-STAI

For the S-STAI scale, we initially reverse scores of the positively connotated items, and then total the scoring weights, resulting in the STAI score (the higher the score the more stressed the participant feels) as per the scoring instructions (https://oml.eular.org/sysModules/obxOML/docs/id_150/State-Trait-Anxiety-Inventory.pdf). To assign some interpretation to the numerical value, we also create a categorical variable, assigning each user to a STAI stress level (i.e., of three levels, (1) below average, (2) average, or (3) above average STAI score). Note that due to human error, the S-STAI scale was administered with a 5-point Likert scale instead of a 4-point one. During processing, we convert each item to a 4-point scale in accordance with the original.

### Privacy considerations

Before the start of the study, we made a commitment to the participants to protect their privacy and sensitive information. Therefore, we thoroughly anonymize the dataset under publication. In the process, we adhere to the following principles: (i) minimizing the probability for successful re-identification of users by real-world adversaries, (ii) maximizing the amount of retained data that are of *use* to the researchers and practitioners, (iii) abiding by the principles and recommendations of GDPR in regards to the handling of personal information, and (iv) following the established anonymization practices and principles.

We strive to maintain *k*-anonymity^22^ of the dataset - ensuring that every user is indistinguishable from at least *k*-1 other individuals. Prior to anonymization, data are stored on secure university servers and proprietary cloud services. According to GDPR, when participants withdraw their consent, their original data are removed, while consent is valid for two years unless withdrawn. Since anonymized data are excluded from the GDPR regulations, they can be stored indefinitely.

We initially remove the identities of the participants such as full names, usernames, and email addresses, and substitute them with a 12-byte random id per user (https://www.mongodb.com/docs/manual/reference/method/ObjectId/). Furthermore, we discard completely (or almost) the parameters that are extremely identifying but are of limited value to the recipients of the dataset, including ethnicity, country, language, and timezone. We also exclude or aggregate some of the physical characteristics of users, namely age, height, weight, and health conditions.

#### Handling quasi-identifiers

By quasi-identifiers we imply non-time-series parameters that can be employed for re-identification. For the anthropometric parameters that cannot be disclosed unmodified, the aggregated versions were released instead. We choose the ranges not just to maintain the anonymity of the dataset but also for them to reflect the real-world divisions. Thus, we split participants into two age categories: below 30, i.e., young adults, and the rest. In a similar manner, we withhold the height and weight of the participants and release BMI instead. To protect the users who may have the outlier values for this parameter, we form 3 distinct BMI groups: < = 19, > = 25, and > = 30 for underweight, overweight, and obese individuals respectively. The participants’ gender (in the context of this study, gender signifies a binary decision that is offered by Fitbit) is the only quasi-identifier that is released unaltered. Based on the quasi-identifiers that are present in the release version of the data (gender, age, and extreme values of BMI) we show that our dataset achieves 2 to 12-anonymity, depending on the “strength” of the adversary we consider.

#### Threat model

To ensure that the anonymity requirements are met, we need to verify that a realistic adversary cannot re-identify the participants. For instance, if the attacker knows that, say, John Smith who participated in the experiment took exactly 12345 steps on a given day, they would easily de-anonymize him. No possible anonymization (except, perhaps, perturbing the time-series data with noise, hence hindering their usability) can preserve privacy in that case.

**Fig. 5:**
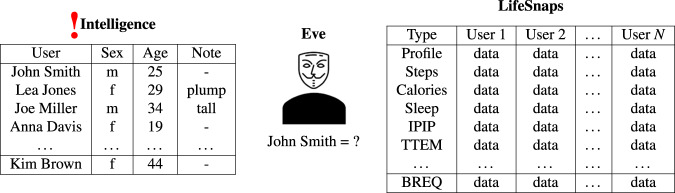
In the threat model we consider, the adversary obtained a list of all the participants in the dataset, found their age, and learned their appearances. They aim to link the individuals (or a single person) back to their aggregated data. Since we do not release height and weight, the physical appearances of the users are significantly less beneficial for de-anonymization. LifeSnaps is 12-anonymous under the normal threat model and at least 2-anonymous under the strongest one.

Instead, we consider a realistic yet strong threat model. Suppose, the adversary obtained the list of all the participants in the dataset and found their birth dates from public sources as depicted in Fig. 5. Moreover, the attacker stalked all the users from the list online or in public places and learned their appearances and some of their distinct traits, e.g., very tall, plump, etc. For the privacy of LifeSnaps to be compromised it is enough for the adversary to de-anonymize just *a single* user. Therefore, we protect the dataset as a whole by ensuring the anonymity of every individual. If the attacker utilizes only the definite quasi-identifiers (gender and age), our dataset achieves unconditional 12-anonymity. Since we do not disclose the height and weight of the participants, the attacker may not directly utilize the observations of physical states (column “Note” in Fig. 5) of the users for de-anonymization. Suppose, the attacker wants to make use of the appearances and associate them with the released BMI. Since BMI is a function of height and weight, they need to estimate both attributes in order to calculate it (Eq. 1). Suppose, the adversary is able to guess the parameters with some error intervals, say, ±5 *kg* for weight and ±5 *cm* for height. The final error interval for BMI can be calculated according to the error propagation (Eq. 2).

1 $$BMI=\frac{weight}{heigh{t}^{2}}$$

2 $${\varepsilon }_{BMI}=\frac{\Delta weight}{weight}+\frac{2\ast \Delta height}{height}$$

For instance, for a person of 170 cm and 70 kg, while $\lfloor BMI\rfloor =24$, the error interval *I* contains 8 integer values, *I* = {21, 22, 23, **24**, 25, 26, 27, 28}. In fact, the list of possible values for BMI stretches nearly from underweight to obese. It is evident that due to the error propagation the adversary is not able to glean significant insights into the users. Even if the attacker is able to *perfectly* estimate the height and weight of the participants, i.e., with no error interval, they can diminish the anonymity factor *k at most* down to 2

## Data Records

### Data access

The LifeSnaps data record is distributed in two formats to facilitate access to broader audiences with knowledge of different technologies, and researchers and practitioners across diverse scientific domains:

1. Through compressed binary encoded **JSON** (BSON) exports of raw data (stored in the mongo_rais_anonymized folder) to facilitate storage in a MongoDB instance (https://www.mongodb.com/);
2. Through **CSV** exports of daily and hourly granularity (stored in the csv_rais_anonymized folder) to facilitate immediate usage in Python or any other programming language. Note that the CSV files are subsets of the MongoDB database presenting aggregated versions of the raw data.

The data are stored in Zenodo (http://zenodo.org/), a general-purpose open repository, and can be accessed online 10.5281/zenodo.6826682^46^. We have compressed the dataset to facilitate usage, but the uncompressed version exceeds 9GB. The data can be loaded effortlessly in a single line of code, and loading instructions are provided in the repository’s documentation. In the remainder of this section, we analyze the structure and organization of the raw data distributed via MongoDB JSON exports.

**Fig. 6:**
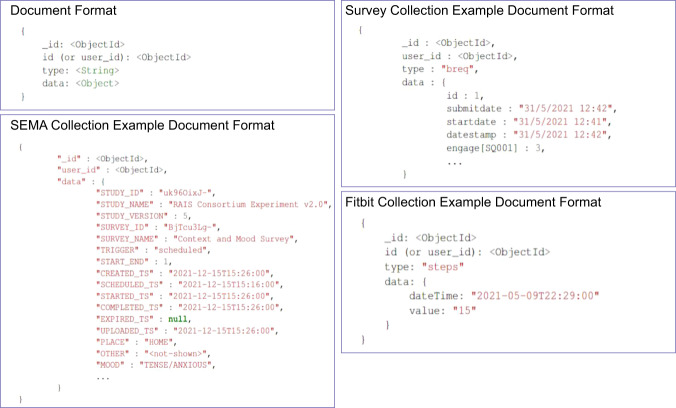
A generic document format for all three collections in MongoDB (top left), along with example document formats for each collection, fitbit, sema, and surveys.

The MongoDB database includes three collections, fitbit, sema, and surveys, containing the Fitbit, SEMA3, and survey data, respectively. Each document in any collection follows the format shown in Fig. 6 (top left), consisting of four fields: _id, id (also found as user_id in sema and survey collections), type, and data. The _id field is the MongoDB-defined primary key and can be ignored. The id field refers to a user-specific ID used to identify each user across all collections uniquely. The type field refers to the specific data type within the collection, e.g., steps (see Table 2 for distinct types per collection). The data field contains the actual information about the document, e.g., steps count for a specific timestamp for the steps type, in the form of an embedded object. The contents of the data object are type-dependent, meaning that the fields within the data object are different between different types of data. In other words, a steps record will have a different data structure (e.g., step count, timestamp, etc.) compared to a sleep record (e.g., sleep duration, sleep start time, sleep end time, etc.). As mentioned previously, all timestamps are stored in local time format, and user IDs are common across different collections.

#### Fitbit collection

**Fig. 7:**
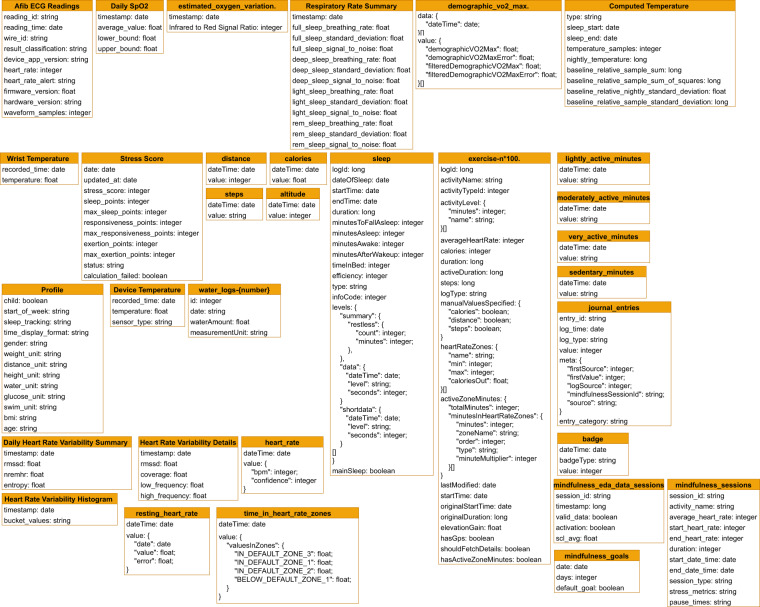
A UML diagram for the Fitbit modality, including all available data types and subtypes.

Regarding the Fitbit collection, it contains 32 distinct data types, such as steps or sleep (see Table 2 for the full list). When it comes to the contents of individual Fitbit data types, researchers should refer to the official company documentation (https://dev.fitbit.com/build/reference/web-api/) for definitions. To assist in future usage, we also provide a UML diagram of the available Fitbit data types and their subtypes in our dataset in Fig. 7. Figure 6 presents the structure of an example document of the fitbit collection (bottom right).

#### Surveys collection

Concerning the survey collection, it contains six distinct data types, namely IPIP, BREQ-2, demographics, PANAS, S-STAI, and TTM. Each type contains the user responses to the respective survey questions (SQ), as well as certain timestamp fields (See the top right document in Fig. 6 for an example BREQ-2 response). Specifically, each SQ is encoded in the dataset for ease-of-use purposes and CSV compatibility. For example, the first SQ of the BREQ-2 scale, i.e., *I exercise because other people say I should*, is encoded as engage[SQ001]. For decoding purposes, we provide tables mapping these custom codes to scale items in the GitHub repository (https://github.com/Datalab-AUTH/LifeSnaps-EDA/blob/main/SURVEYS-Code-to-text.xlsx) and Zenodo. User responses to SQs are shared as is without any encoding. Each survey document also contains three timestamp fields, namely “submitdate” (i.e., timestamp of form submission), “startdate” (i.e., timestamp of form initiation), and “datestamp” (i.e., timestamp of the last edit in the form after submission; coincides with the “submitdate” if no edits were made). To facilitate usage, in the folder scored_surveys, we provide scored versions of the raw survey data in CSV format, as discussed in the Data Preprocessing section.

#### SEMA collection

Finally, regarding the sema collection, it contains two distinct data types, namely Context and Mood Survey, and Step Goal Survey. Each EMA document contains a set of fields related to the study and the survey design (e.g., “STUDY_NAME”, “SURVEY_NAME”), as well as the participant responses to the EMA questions, and timestamp fields. Figure 6 (bottom left) presents an example of the “Context and Mood Survey”, where the participant is at home (i.e., “PLACE”: “HOME”) and they are feeling tense (i.e., “MOOD”: “TENSE/ANXIOUS”). Apart from the pre-defined context choices, participants are also allowed to enter their location as a free text, which appears in the “OTHER” field. Concerning the timestamp fields, each document contains the following: “CREATED_TS” (i.e., the timestamp from when the survey was created within the SEMA3 app), “SCHEDULED_TS” (i.e., the timestamp from when the survey was scheduled, e.g., when the participant received the notification), “STARTED_TS” (i.e., the timestamp from when the participant started the survey), “COMPLETED_TS” (i.e., the timestamp from when participant completed the survey), “EXPIRED_TS” (i.e., the timestamp from when the survey expired), “UPLOADED_TS” (i.e., the timestamp from when the survey was uploaded to the SEMA3 server). Note that if a participant completes a scheduled EMA, then the EXPIRED_TS field is null, otherwise, the COMPLETED_TS field is null. Only completed EMAs have valid values in the STEPS field for the Step Goal Survey or the PLACE and MOOD fields for the Context and Mood Survey.

### Use cases

**Fig. 8:**
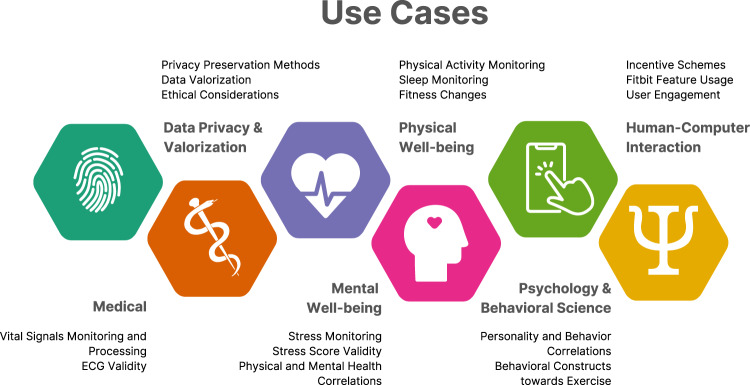
Indicative use cases for the LifeSnaps dataset.

We built the LifeSnaps Dataset with the goal to serve multiple-purpose scientific research. In this section, we discuss indicative use cases for the data (see Fig. 8), and hope that researchers and practitioners will devise further uses for various aspects of the data.

Among others, the LifeSnaps dataset includes data emerging from diverse vital signals, such as heart rate, temperature, and oxygen saturation. Such signals can be of use to *medical* researchers and practitioners for general health monitoring, but also to *signal processing* experts due to their fine granularity. When it comes to coarse granularity data, the dataset includes a plethora of physical activities, sleep sessions, and other data emerging from behaviors related to physical well-being. Such data can be exploited by researchers and practitioners within the sleep research and sports sciences domain to study how human behavior affects overall *physical well-being*. On top of that, the LifeSnaps dataset is a rich source of mood and affect data, both measured and self-reported, that have the potential to empower research in the domains of stress monitoring and prediction, and overall *mental well-being*. Additionally, the diverse modalities of the dataset allow for exploring the correlation between objectively measured and self-reported mental and physical health data, while the psychological and behavioral scales distributed can facilitate research in the *behavioral sciences* domain. On a different note, incentive schemes, such as badges, and their effect on user behavior, user compliance, and engagement with certain system features could be of great interest to *human-computer interaction* experts. Finally, handling such sensitive data can fuel discussions and efforts towards *privacy* preservation research and *data valorization*.

## Technical Validation

Fitbit data validity has been extensively studied and verified in prior work^47–49^. To this end, this section goes beyond data validity to discuss data quality issues emerging from the study procedure, as presented in the previous section. Specifically, we provide details on user engagement and compliance with the study, and we delineate data completeness and other limitations that we have encountered during the process.

### User engagement

#### Surveys

**Fig. 9:**
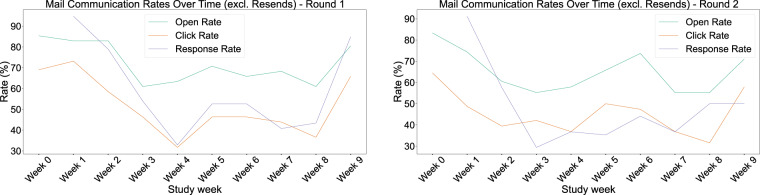
Mail communication open and click rates (excluding resent e-mails) and response rates (overall) for first (left) and second (right) study round.

Throughout both study rounds, the users received reminder e-mails, as discussed earlier, for weekly survey completion. The open and click rates after e-mail communication vary, as shown in Fig. 9. Overall, during the first round, the compliance is higher than that of the second, while in both study rounds, there is a sharp decrease between weeks 2–4 concerning open, click, and response rates. The open and click rates follow approximately the same trend and they exhibit a high percentage (~80% and ~65%, respectively) in the beginning and end of the study batches and a medium percentage between weeks 5–7 (~70% and ~50%, respectively). The response rate shows a steep drop from week 1 to week 3–4 in both rounds plummeting almost to 30% at its lowest level (from more than 90% in the beginning), and settling around 40–50% for the second month of each round. Note that the response rate is sometimes higher than the open or click rates, due to resent reminder e-mails (discussed earlier in the data collection monitoring section). An interesting finding is that reminder e-mails seem to be more effective in the first weeks of the study, where the response rate is higher than the open and click rates, but have diminished effectiveness later on, where the response rate is similar to the click rate excluding resents.

#### SEMA3

**Fig. 10:**
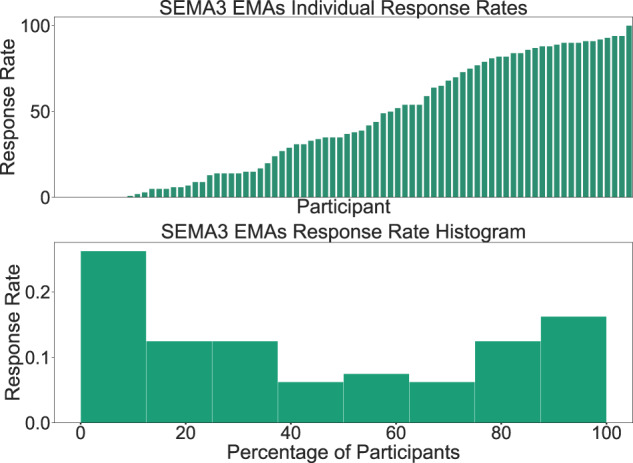
SEMA3 EMAs response rate bar plot (up) and histogram (bottom) for both rounds of the experiment.

**Fig. 11:**
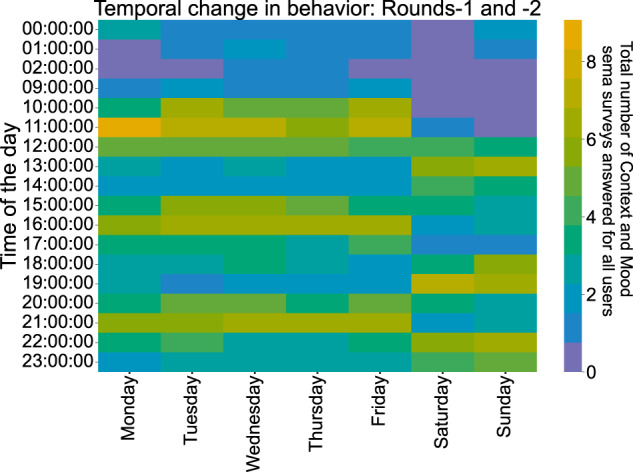
When did our participants answer the SEMA3 EMAs?.

We also study and quantify user engagement through the lens of the SEMA3 EMA responses. The SEMA3 average user compliance for both rounds is 43%, calculated from the SEMA3 online platform. Moreover, Fig. 10 shows the different compliance levels in terms of response rate across participants. Contrary to related studies of a similar scale^17^, where there is a significant decline in the number of users responding to the daily and weekly surveys, and the response rate distribution closely resembles an exponential decay curve, in our experiment, nearly 1 in 3 participants replied to more than 75% of the EMA surveys (bottom subfigure). In the top subfigure, we also notice that numerous participants exhibited extremely high levels of engagement (>90%). Additionally, Fig. 11 depicts when the participants preferred to answer the SEMA3 EMAs surveys throughout the week. It seems that the most common time slot was during the weekdays between 10 to 12 am.

**Fig. 12:**
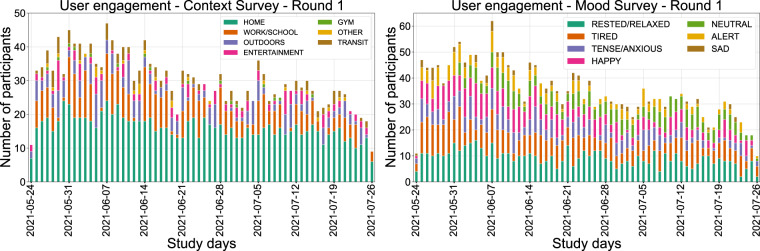
Context (left) and mood (right) EMA responses per day for the first study round. Every date shown in the figure is a Monday.

**Fig. 13:**
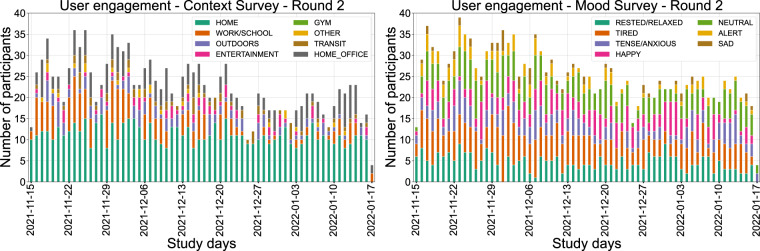
Context (left) and mood (right) EMA responses per day for the second study round. Every date shown in the figure is a Monday.

Regarding the completeness of the SEMA3 responses, participants engaged in various activities throughout their daily routine during the study. The Context and Mood EMA responses (Figs. 12 and 13) give us a peek into the participants’ lifestyles, given that data are collected in the wild. During both rounds, the most frequent response to where our participants were located was *HOME*, which is unsurprising during the Covid-19 pandemic, with the second most frequent response being either *WORK* or *SCHOOL*. During the first study round, the answer *OUTDOORS* appears more often than in the second one, which might be partly explained because the first round occurred during summer in Europe, when the weather is more friendly for outdoor activities. Regarding emotions, interestingly, *TIREDNESS* is a response that usually appears at the beginning of the week and *SADNESS* appears on Mondays or in the middle of the week. On the same note, the participants seem to use *HAPPY* more often on Fridays or during the weekends. *HAPPY* was also selected an outstanding amount of times during Christmas vacations.

#### Fitbit

**Fig. 14:**
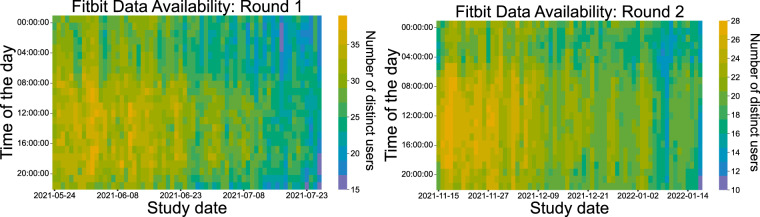
Fitbit data availability throughout the first (left) and second (right) study round.

**Fig. 15:**
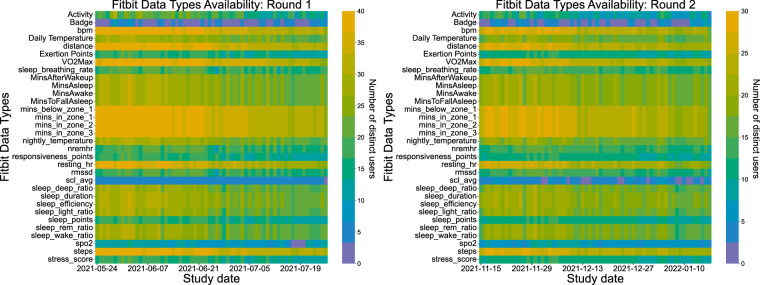
All Fitbit data types available throughout the first (left) and second (right) study rounds.

Having discussed user engagement with regard to surveys and EMAs, from here onward we explore compliance with the Fitbit device. The heatmaps in Fig. 14 display the Fitbit data availability for both rounds, incorporating the distance, steps, and estimated oxygen variation Fitbit types, while the ones in Fig. 15 depict all available Fitbit Types throughout the study dates of both study rounds. Although there is a declining trend throughout the study dates in both study batches, the participants’ engagement is still outstanding, producing valuable data as the mean user engagement rises to 41.37 days (42.71 days in the first round and 40.03 in the second round), corresponding to 73% of the total study days. The standard deviation is 16.67 days and the median is 49.5 days in the first round, while in the second round the standard deviation and median are 17.39 and 40.13 days respectively. An interesting finding is a decline in Fitbit usage in the first days of the year (second round), and a resumption to normal use shortly after. We also notice that the number of users who wear their Fitbit device during the night is smaller compared to during the daytime, which is in accordance with prior work, revealing that almost 1 out of 2 users do not wear their watch during sleeping or bathing^50^.

**Fig. 16:**
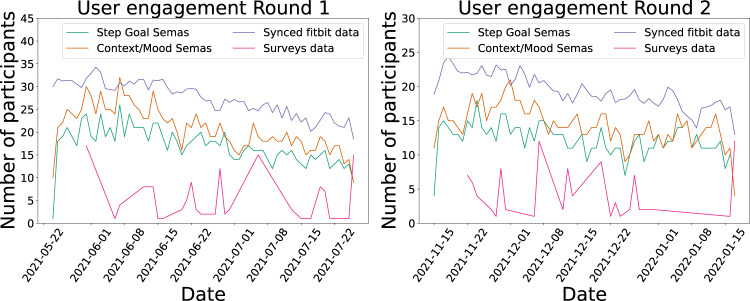
User engagement (Fitbit, SEMA3 and Surveys collections) throughout the first (left) and second (right) study round.

Summing up, in Fig. 16, all collection types, namely the synced Fitbit data, the Step Goal and Context and Mood SEMA3 questionnaires, and the surveys data are compared, visualizing the complete picture of the user engagement throughout both rounds. In both study batches, we did not lose a significant number of participants from the beginning to the end, contributing to the completeness and quality of the LifeSnaps dataset. Note that while Fitbit and SEMA3 engagement waned with time, survey participation peaked at the end of the study. We presume this is due to the repeated e-mails unresponsive participants received for extracting and sharing their Fitbit data accompanied by survey completion reminders. It is worth mentioning that the participants could respond to the surveys throughout the week after receiving the notification e-mail, which is why the survey data appear more spread and low through the study dates during both study rounds.

**Fig. 17:**
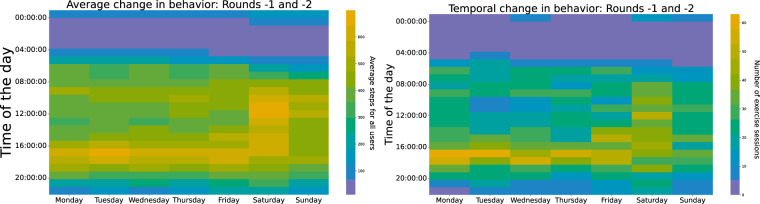
Pattern analysis: When and how much have the participants walked (left) and exercised (right).

Digging a little bit into the data, we can visualize (Fig. 17) the average steps (left) and the number of exercise sessions (right) for all participants combined for both study batches. We can clearly see that there is a significant increase in the number of steps around 5PM during the weekdays assuming that most people were leaving their jobs at that time. As expected, the steps start to appear later in the morning during the weekends compared to the weekdays and Saturday seems to be the day of the week with the largest mean number of steps. Regarding the time and day of the week, our participants preferred to exercise, on Mondays and Saturdays the sessions appear more spread throughout the daytime. Similarly, with the steps pattern, there is a rise in the number of exercise sessions starting from 5PM during the weekdays and an outstanding elevation on Saturdays.

Note here that the majority of published datasets do not provide information on user engagement. On the contrary, we discuss user engagement patterns for all three modalities (i.e., Fitbit, SEMA, and surveys) separately, reaffirming the quality and validity of the LifeSnaps dataset. Additionally, according to Yfantidou *et al*.^51^, 70% of health behavior change interventions (user studies) utilizing self-tracking technology have a sample size of fewer than 70 participants, while 59% has a duration of fewer than 8 weeks. In accordance with these findings, the median sample size of the relevant studies discussed in the introduction is a mere 16 users, while a significant number of these datasets are constrained to lab conditions. Such comparisons highlight the research gap we aim to close and the LifeSnaps dataset’s importance for advancing ubiquitous computing research.

### Limitations

The LifeSnaps study experienced similar limitations to previous personal informatics data collection studies both in terms of participant engagement and technical issues.

As discussed earlier, while participant dropout rates are low (7%), there exists a decreasing trend in compliance over time across data modalities. Despite the data collection monitoring system in place and the subsequent nudging of non-compliant participants, we still encountered a ~18% decrease in SEMA3 compliance (~24% in the first round and ~11% in the second round), ~18% decrease in Fitbit compliance (~24% in the first round and ~12% in the second round), and approximately 5% decrease in survey communication open rates between entry and exit weeks. Note here that a small percentage of non-compliant participants with Fitbit is due to allergic reactions participants developed to the watch’s wristband and reported to the research team. At the same time, waning compliance with the smartwatch is expected due to well-documented novelty effects lasting from a few hours to a few weeks^52^. Nevertheless, such compliance rates are still higher than similar studies of the same duration^53^. To better understand how non-compliant participants can be engaged, differing incentive mechanisms should be put in place to gauge how such participants would respond.

Independent of compliance issues, we also encountered a set of technical limitations that were out of our control:

#### Device incompatibility

One participant (~1%) reported full incompatibility of their mobile phone device with the SEMA3 app. To decrease entry barriers, we did not restrict accepted operating systems for the users’ mobile phones. Despite SEMA3 supporting both Android and iOS, there are known limitations with regard to certain mobile phone brands (https://docs.google.com/document/d/1e0tQvFSnegpJg2P5mfMU7N7R9D3gTZatzxGmVy-EkQE/edit). Additionally, multiple participants reported issues with notifications and SEMA3 operation. All received troubleshooting support in accordance with the official manual above. There were no compatibility issues reported for the Fitbit device.

#### Corrupted data

While processing the data, we encountered corrupted data records due to either user or system errors. For instance, there exists a user weighing 10 kg, which is an erroneous entry that also affects the calculation of correlated data types such as calories. Concerning system errors, we noticed corrupted dates (e.g., 1 January 1980 00:00:00) for a single data type in the surveys collection, which emerged from the misconfiguration of the LimeSurvey proprietary server. Also, we encountered outlier data values (e.g., stress score of 0) outside the accepted ranges. We leave such entries intact since they are easily distinguishable and can be handled on a case-by-case basis. Finally, we excluded swimming data because they portrayed an unrealistic picture of the users’ activity, i.e., false positives of swimming sessions in an uncontrolled setting, possibly due to issues with the activity recognition algorithm.

#### Missing data

Missing Fitbit data emerge either from no-wear time or missing export files. No-wear time is related to participants’ compliance and has been discussed previously (see Figs. 14 and 15). Missing export files most likely emerge from errors in the archive export process on the Fitbit website and are outside of our control. For instance, while we have resting heart rate data for 71 users, we only have heart rate data -the most common data type- for 69 users (~3% missing). Similarly, as seen in Table 2, we encountered unexpectedly missing export files for the steps (~3%), profile (~1%), exercise (~3%), and distance (~4%) data types. Missing SEMA3 and survey data emerge solely from non-compliance.

#### External influences

We encouraged the participants to continue their normal lives during the study without scripted tasks. Such an approach embraces our study’s “in the wild” nature but is more sensitive to external influences. For instance, we assume a single time zone per participant (taken from their Fitbit profile), as described in our methodology. However, if a participant chooses to travel to a location with a different time zone during the study, this assumption no longer holds. Based on our observations from the SEMA3 provided time zones, this only applies to ~3% of participants and for a limited time period. Additionally, the study took place during the Covid-19 pandemic and its related restrictions, which might have affected the participants’ behavior. However, we see this external influence as an opportunity for further research rather than a limitation.

#### Lack of documentation

While Fitbit provides thorough documentation for its APIs, there is no documentation regarding the archive export file format, with the exception of the integrated README files. To this end, it was impossible to define the export time zone per Fitbit data type without analysis. To overcome this challenge, we compared record-by-record the exported data with the dashboard data on the Fitbit mobile app, which are always presented in local time. Table 3 is the result of this comparison.

## Usage Notes

While we provide the LifeSnaps dataset with full open access to “enable third parties to access, mine, exploit, reproduce and disseminate (free of charge for any user) this research data” (https://ec.europa.eu/research/participants/docs/h2020-funding-guide/cross-cutting-issues/open-access-data-management/open-access_en.htm), we encourage everyone who uses the data to abide by the following code of conduct:

- You confirm that you will not attempt to re-identify the study participants under no circumstances;
- You agree to abide by the guidelines for ethical research as described in the ACM Code of Ethics and Professional Conduct^54^;
- You commit to maintaining the confidentiality and security of the LifeSnaps data;
- You understand that the LifeSnaps data may not be used for advertising purposes or to re-contact study participants;
- You agree to report any misuse, intentional or not, to the corresponding authors by mail within a reasonable time;
- You promise to include a proper reference on all publications or other communications resulting from using the LifeSnaps data.

## Data Availability

All code for dataset anonymization is available at https://github.com/Datalab-AUTH/LifeSnaps-Anonymization. All code for reading, processing, and exploring the data is made openly available at https://github.com/Datalab-AUTH/LifeSnaps-EDA. Information about setup, code dependencies, and package requirements are available in the same GitHub repository.
